# A Study of Factors Associated With Melasma and Quality of Life in Patients With Melasma: A Case-Control Study

**DOI:** 10.7759/cureus.103331

**Published:** 2026-02-10

**Authors:** Ram H Malkani, Sonar Narula, Chander Lulla, Suman Karmakar, Jekin Choubisa, Anjali Sharma, Maninder S Setia

**Affiliations:** 1 Dermatology, Jaslok Hospital and Research Centre, Mumbai, IND; 2 Laboratory Medicine, Microbiology, Jaslok Hospital and Research Centre, Mumbai, IND; 3 Radiology, Jaslok Hospital and Research Centre, Mumbai, IND; 4 Clinical Research, Dr Skin Pimples Pvt Ltd, Mumbai, IND; 5 Epidemiology, Mahatma Gandhi Mission (MGM) Institute of Health Sciences, Navi Mumbai, IND

**Keywords:** factors, gender, melasma, metabolic syndrome, quality of life

## Abstract

Introduction

Melasma is a common condition seen in dermatology clinics and is considered to be a multifactorial disease. We designed the present study to: study the factors associated with melasma (including metabolic syndrome (MetS) and biochemical parameters); compare the characteristics of melasma and other factors (MetS and biochemical parameters) in male and female melasma patients; and evaluate the quality of life in melasma patients and its correlation with the severity of the condition.

Methods

This study is a case-control study of 80 individuals with melasma and 80 controls attending a private dermatology clinic in Mumbai, India. We collected demographic details and other risk factors in both groups and clinical details in patients with melasma. We assessed the following biochemical parameters, such as fasting blood sugar, glycated hemoglobin (HbA1c), triglycerides, and high-density lipoproteins. In patients with melasma, severity was evaluated using the Melasma Area and Severity Index (MASI) score, and quality of life using the Melasma Quality of Life (MELASQOL) questionnaire.

Results

The mean (SD) age of the cases (37.2 (5.9)) was significantly higher than controls (30.6 (7.0)) years (p<0.001). A significantly higher proportion of cases had a family history (in first-degree relatives) compared with controls (52.5% (n=42) vs 16.3% (n=13); p<0.001). A lower proportion of cases were classified as MetS compared with controls; however, it was not statistically significant (30.0% (n=24) vs 33.8% (n=27); p=0.61). Although a higher proportion of females had metabolic syndrome compared with males, the difference was not statistically significant (35.2% (n=19) vs 19.2% (n=5); p=0.15). In multivariate models, melasma was significantly associated with age ≥35 years (odds ratio (OR): 4.3, 95% confidence interval (CI): 1.6, 11.4; p<0.01), being married (OR: 4.8, 95% CI: 1.1, 21.7; p<0.01), and a family history of melasma (OR: 8.1, 95% CI: 2.9, 23.1; p<0.01). Males melasma cases were more likely to have high triglyceride levels compared with females (OR: 8.6, 95% CI: 1.6, 47.5; p=0.014). Correlation between MASI and MELASQOL scores was statistically significant in females (r*=*0.28; p=0.04), but not in males (r*=*12; p=0.573).

Conclusions

Factors associated with melasma were age ≥35 years, a family history of melasma, and being married. There was no significant difference in those classified as metabolic syndrome between these two groups. In melasma cases, in general, there was no significant difference in demographic and clinical characteristics between males and females. However, males had significantly higher levels of triglycerides compared with females. The association between melasma severity and quality of life was significant only in females.

## Introduction

Melasma is a common condition seen in dermatology clinics. It has been estimated that women outnumber male patients attending the clinic for melasma in a ratio of 4:1 to as high as 9:1 making it an important dermatological and cosmetic condition [1-4]. The prevalence of melasma varies from as low as 1.5% to as high as 15.5% across multiple groups globally [5-8]. The proportion of female melasma patients is higher in dermatology clinical settings compared with males; however, the prevalence is higher in pregnancy and may vary from 16% to 51% in different populations [9,10]. Melasma is often considered to be a multifactorial disease. Common associations include exposure to sunlight and a family history of melasma. Studies have shown that the duration of sun exposure and latitude may be associated with the prevalence, exacerbation, and severity of melasma [11-15]. Family history and genetic inheritance have also been reported as factors for the occurrence of melasma [14,16,17]. Other factors considered in melasma include hormones, such as estrogen and progesterone, which in predisposed persons may act synergistically with ultraviolet B and lead to hyperpigmentation [14,16,18-20]. Additionally, oral contraceptives and autoimmunity, particularly thyroid disorders, have also been reported as factors associated with melasma [21-23].

Recently, there has been discussion on the role of metabolic factors, including metabolic syndrome (MetS), in cutaneous pigmentation [24]. Some authors have compared lipid parameters in melasma patients and controls, while others have suggested that we should be cautious when interpreting the link between lipid metabolism and the pathogenesis of melasma [25-27]. Kang and colleagues reported downregulation of lipid-metabolism-associated genes in melasma-lesional skin in bioinformatics analysis [28]. Other authors have shown that oxidative parameters, oxidative stress, and other metabolites may be altered in patients with melasma [29-32]. Some authors have also suggested that insulin resistance may be associated with skin pigmentation and encourage further studies in this area [33]. Therefore, there may be a need to understand the metabolic parameters along with other associated factors in melasma. Since melasma is a pigmentary condition and usually visible, it may also affect the quality of life in these patients [34]. It is important to understand the correlation between melasma-related parameters and the quality of life. Studies have described the factors in male and female melasma patients, but it is also important to understand the differences in the quality of life in male and female patients [35-37].

With this background, we designed the present study to: study the factors associated with melasma (including MetS and biochemical parameters); compare the characteristics of melasma and other factors (MetS and biochemical parameters) in male and female melasma patients; and assess the quality of life in melasma patients and its correlation with the severity of the condition.

## Materials and methods

This present study is a case-control study of 80 individuals with melasma and 80 controls attending a dermatology clinic in Mumbai, India.

Study site and population

The study was conducted in a tertiary care private hospital in Mumbai, India. All the patients who attended the Dermatology clinic of this hospital were eligible for inclusion in the study, either as a case or a control. Patients aged 18 years and above with pigmentation on the face and diagnosed with melasma were considered as potential cases. All patients who were on medications that could exacerbate facial pigmentation were excluded from the study. We also excluded pregnant women and those who were on oral contraceptive pills currently or in the past six months. Individuals (≥18 years) without any facial pigmentation suggestive of melasma at present or in the past were considered as controls. All consecutive consenting patients who met the inclusion criteria were included in the study either as a case or a control.

Study procedures

A detailed questionnaire was administered to all the patients included in the study. The questionnaire included the following components: (1) demographic data (age, gender, marital status, occupation, income); (2) detailed history of melasma to cases (such as onset, duration, progress, treatment taken, family history-first-degree relatives); (3) use of oral contraceptive pills and menstrual history (in females); (4) sun exposure and sun screen use; and (5) other systemic co-morbidities (such as diabetes mellitus or thyroid disorders).

We also measured the weight, height, body mass index, and systolic and diastolic blood pressure of these individuals. Blood samples were collected for measuring the following biochemical parameters: fasting blood sugar, glycated hemoglobin (HbA1c), triglycerides, and high-density lipoproteins. Participants with abnormal values were referred for clinical evaluation and management.

For melasma cases, detailed clinical examination was conducted, including a Wood's lamp examination, Melasma Area and Severity Index score (MASI), and quality of life assessment using the Melasma Quality of Life (MELASQOL) questionnaire [38-42]. We used the International Diabetes Federation criteria for the classification of patients as metabolic syndrome (MetS) in our study [43]. Finally, an ultrasonography examination was performed on female cases and controls to evaluate for polycystic ovaries or other features.

Statistical methods

Data were entered in Microsoft Excel (Microsoft Corporation, Redmond, WA, USA) and converted to Stata Version 17 (StataCorp©, College Station, Texas, USA) for analysis.

We estimated the means and standard deviations (SDs) or median and interquartile range for linear variables. We estimated the proportions for categorical variables. The means across groups were compared using the unpaired t-test for normally distributed data. For data that were not normally distributed, we used the Wilcoxon-Mann-Whitney rank-sum test. The proportions were compared using the chi-square test or Fisher's exact test for low expected cell counts. The correlation between two linear variables was assessed using Pearson's correlation co-efficient (r). We used logistic regression models for multivariate analysis of data for categorical variables and linear regression models for linear data. The fit of the models and variables in the model was assessed using Akaike Information Criteria, Bayesian Information Criteria, and Variance Inflation Factor [44-46]. A p-value of <0.05 was considered statistically significant.

The study was approved by the Institutional Ethics Committee of Jaslok Hospital (Project Reference Number 913). The study was conducted in accordance with the principles of the Declaration of Helsinki and Good Clinical Practices. All participants provided a written informed consent prior to inclusion in the study.

## Results

The present study included 80 cases of melasma and 80 controls. The mean (SD) age of the cases (37.2 (5.9)) was significantly higher than that of controls (30.6 (7.0)) years (p<0.001). There was no significant difference in the proportion of males and females between cases and controls. A higher proportion of melasma cases were married compared with controls (88.8% (n=71) vs 60.0% (n=48)); there was a significant difference in marital status between cases and controls (p<0.001). A significantly higher proportion of controls had no children compared with cases (Table 1). The proportion of socio-economic categories did not differ significantly between melasma cases and controls (p=0.88). A higher proportion of cases reported family history (in first-degree relatives) compared with controls (52.5% (n=42) vs 16.3% (n=13)). There was a significant difference in the number of children between cases and controls (p<0.001). There was no significant difference in the mean (SD) BMI of cases and controls (25.6 (5.7) vs 26.4 (6.2) kg/m^2^; p=0.40). Although a higher proportion of controls reported sun exposure of less than one hour per day (62.5% (n=50) vs 47.5% (n=47.5)), there was no significant difference in the sun exposure between cases and controls (p=0.16). A higher proportion of cases reported use of sunscreens compared with controls (33.8% (n=27) vs 20.0% (n=16); p=0.05). Only one participant in the control group had a history of diabetes mellitus. The proportion of individuals reporting thyroid disorders (hypo/hyperthyroidism) was higher in the case group compared with the control group (6.3% (n=5) vs 0% (n=0); p=0.059); though the difference was not statistically significant. Only three females gave a past history of using oral contraceptive pills; the proportion was not significantly different between cases and controls (3.7% (n=2) vs 2.5% (n=1); p=0.74). Complete details are presented in Table 1.

**Table 1:** Table showing select demographic, clinical, and sun exposure characteristics of 80 patients with melasma and 80 without any melasma, Mumbai, India. SD: standard deviation, IQR: interquartile range, MASI: Melasma Area and Severity Index score, MELASQOL: Melasma Quality of Life. *The N for controls was 79 (one missing value). The percentages are row percentages for the total and row percentages for the parameters. The p-values were calculated using the chi-square test or Fisher's exact test for low expected cell counts.

Characteristics	Total	Melasma	Control	p-value
All	160 (100)	80 (50)	80 (50)	
Age groups (years)				
18-34	81 (50.6)	24 (30.0)	57 (71.3)	<0.001
≥35	79 (49.4)	56 (70.0)	23 (28.7)	
Gender				
Male	66 (41.3)	26 (32.5)	40 (50.0)	
Female	94 (58.8)	54 (67.5)	40 (50.0)	0.025
Marital status				
Never married	40 (25.0)	8 (10.0)	32 (40.0)	<0.001
Married	119 (74.4)	71 (88.8)	48 (60.0)	
Divorced/separated	1 (0.6)	1 (1.3)	0 (0)	
Socio-economic status				
Upper middle	60 (37.5)	28 (35.0)	32 (40.0)	0.88
Lower middle	52 (32.5)	28 (35.0)	24 (30.0)	
Upper lower	31 (19.4)	15 (18.8)	16 (20.0)	
Lower	17 (10.6)	9 (11.3)	8 (10.0)	
Children*				
None	60 (37.7)	18 (22.5)	42 (53.2)	<0.001
One	36 (22.6)	18 (22.5)	18 (22.8)	
≥2	63 (39.6)	44 (55.0)	19 (24.0)	
Family history (first-degree relatives)				
No	108 (67.5)	38 (47.5)	67 (83.7)	<0.001
Yes	55 (34.4)	42 (52.5)	13 (16.3)	
Body mass index				
Mean (SD)	26.0 (5.9)	25.6 (5.7)	26.4 (6.2)	0.40
Weight categories				
Underweight	16 (10.0)	8 (10.0)	8 (10.0)	0.15
Normal	57 (35.6)	35 (43.8)	22 (27.5)	
Overweight	60 (37.5)	24 (30.0)	36 (45.0)	
Obese	27 (16.9)	13 (16.3)	14 (17.5)	
Sun exposure (daily)				
Less than one hour	88 (55.0)	38 (47.5)	50 (62.5)	0.16
1-2 hours	44 (27.5)	26 (32.5)	18 (22.5)	
>2 hours	28 (17.5)	16 (20.0)	12 (15.0)	

There was no significant difference in the serum triglyceride and high-density lipoprotein values between cases and controls (Table 2). There was no significant difference in fasting blood sugar and HbA1c levels between the cases and controls. A lower proportion of cases were classified as MetS compared with controls; however, the difference was not statistically significant (30.0% (n=24) vs 33.8% (n=27); p=0.61). Complete details are presented in Table 2.

**Table 2:** Table showing select biochemical parameters and metabolic syndrome in 80 cases of melasma and 80 controls, Mumbai, India. IDF: International Diabetes Federation, Hb1Ac: glycated hemoglobin, IQR: interquartile range, SD: standard deviation. The details for the estimate are shown in the table. The p-values were calculated using an unpaired t-test for parametric data, a Wilcoxon-Mann-Whitney test for non-parametric data, and a chi-square test for proportions.

Characteristics	Total	Melasma	Control	p-value
Total, N (%)	160 (100)	80 (50)	80 (50)	
Serum triglycerides (mg/dl), median (IQR)	100.7 (67.5, 138.5)	92.8 (65.3, 137.0)	111.4 (69.5, 139.5)	0.51
Serum high-density lipoproteins (mg/dl), median (IQR)	43.0 (37.5, 49.5)	43.6 (37.6, 50.0)	42.6 (37.3, 48.9)	0.43
Fasting blood sugar (mg/dl), mean (SD)	94.3 (13.5)	94.1 (8.9)	94.6 (16.9)	0.83
HbA1c (%), mean (SD)	5.43 (0.65)	5.44 (0.62)	5.43 (0.67)	0.97
Metabolic syndrome IDF definition, n (%)	51 (31.9)	24 (30.0)	27 (33.8)	0.61

When comparing the melasma features between males and females, we did not find any significant difference in terms of duration, progress of melasma, family history, or treatment taken for melasma. Although family history was common in females compared with males, the difference was not statistically significant (53.7% (n=29) vs 50.0% (n=13); p=0.76). There was a significant difference in the number of children between males and females (Table 3). The median (IQR) MASI score was lower in males compared with females; however, the difference was not statistically significant (3.25 (2.70, 5.70) vs 3.45 (2.10, 8.10); p=0.77). The median (IQR) Melasma QOL score was lower in males compared with females (16 (13, 26) vs 19 (12, 37); p=0.46); however, the difference was not statistically significant. We have presented detailed findings in Table 3.

**Table 3:** Table showing the clinical characteristics and demographic characteristics of male and female melasma patients, Mumbai, India. SD: standard deviation, IQR: interquartile range, MASI: Melasma Area and Severity Index score, MELASQOL: Melasma Quality of Life. *Also includes the single case of divorce. The percentages are row percentages for the total and row percentages for the parameters. The p-values were calculated using the chi-square test or Fisher's exact test for low expected cell counts.

	Total population	Males	Female	p-value
	N (%)	n (%)	n (%)	
Total	80 (100)	26 (32.5)	54 (67.5)	
Age groups				
18-34	24 (30.0)	10 (38.5)	14 (25.9)	0.25
≥35	56 (70.0)	16 (61.5)	40 (74.1)	
Marital history				
Single	8 (10.0)	5 (19.2)	3 (5.6)	0.06
Married*	72 (90.0)	21 (80.8)	51 (94.4)	
Number of children				
None	18 (22.5)	9 (34.6)	9 (16.7)	0.01
One	18 (22.5)	9 (34.6)	9 (16.7)	
≥2	44 (55.0)	8 (30.8)	36 (66.6)	
Socio-economic status				
Upper middle	28 (35.0)	8 (30.8)	20 (37.0)	0.45
Lower middle	28 (35.0)	12 (46.2)	16 (29.6)	
Upper lower	15 (18.8)	3 (11.5)	12 (22.2)	
Lower	9 (11.3)	3 (11.5)	6 (11.1)	
Family history (first-degree relatives)				
No	38 (47.5)	13 (50.0)	25 (46.3)	0.76
Yes	42 (52.5)	13 (50.0)	29 (53.7)	
Body mass index				
Mean (SD)	25.6 (5.8)	24.0 (3.0)	26.4 (6.6)	0.08
Duration of melasma (months)				
Median (IQR)	18 (12, 42)	21 (12, 48)	18 (12, 36)	0.71
Progress				
Increased with time	63 (78.8)	44 (81.5)	19 (73.1)	0.64
Decreased with time	3 (3.8)	2 (3.7)	1 (3.9)	
No change	14 (17.5)	8 (14.8)	6 (23.1)	
MASI score				
Median (IQR)	3.40 (2.40, 6.45)	3.25 (2.70, 5.70)	3.45 (2.10, 8.10)	0.77
Treatment taken				
Only topical	18 (22.5)	8 (30.8)	10 (18.5)	0.22
Topical and systemic	15 (18.8)	4 (15.4)	11 (20.4)	0.59
None	47 (58.8)	14 (53.1)	33 (61.1)	0.54
MELASQOL score				
Median (IQR)	16.5 (13, 33.5)	16 (13, 26)	19 (12, 37)	0.46

The ultrasonography findings were as follows: melasma cases-polycystic ovary features (20.4% (n=11)), multifocal ovary (3.7% (n=2)), adenomyosis (3.7% (n=2)); and controls-polycystic ovary features (42.5% (n=17)), adenomyosis (2.5% (n=1)); the difference in proportions was not statistically significant (p=0.063). The majority of the females reported regular menstrual cycles (77.8% (n=42)) and only 16.7% (n=9) had irregular menstrual cycles. We also compared the biochemical parameters in male and female melasma patients. The median (IQR) serum triglyceride levels (mg/dl) were significantly higher in males compared with females (131.5 (84.5, 173.3) vs 82.4 (63.0, 111.2); p=0.003). A higher proportion of males had high triglyceride levels (≥150 mg/dl) compared with females (38.5% vs 12.9%; p=0.009). Even though a higher proportion of females had metabolic syndrome compared with males, the difference was not statistically significant (35.2% (n=19) vs 19.2% (n=5); p=0.15) (Table 4).

**Table 4:** Table showing select biochemical parameters and metabolic syndrome in male and female cases of melasma, Mumbai, India. IDF: International Diabetes Federation, HbA1c: glycated hemoglobin, IQR: interquartile range, SD: standard deviation. The details for the estimate are shown in the table. The p-values were calculated using an unpaired t-test for parametric data, a Wilcoxon-Mann-Whitney test for non-parametric data, and a chi-square test for proportions.

Characteristics	Females	Males	p-value
Total N (%)	54 (67.5)	26 (32.5)	
Serum triglycerides (mg/dl), median (IQR)	82.4 (63.0, 111.2)	131.5 (84.5, 173.3)	0.003
Serum high-density lipoproteins (mg/dl), median (IQR)	44.9 (37.3, 50.7)	41.4 (39.1, 48.3)	0.31
Fasting blood sugar (mg/dl), mean (SD)	93.2 (8.2)	95.9 (10.2)	0.19
HbA1c (%), mean (SD)	5.48 (0.69)	5.35 (0.43)	0.38
Metabolic syndrome IDF definition, n (%)	19 (35.2)	5 (19.2)	0.15

In the unadjusted logistic regression models, the factors associated with melasma were age ≥35 years (odds ratio (OR): 5.8, 95% confidence interval (CI): 2.9, 11.4; p<0.01), female gender (OR: 2.1, 95% CI: 1.1, 3.9; p<0.05), being married (OR: 6.0, 95% CI: 2.6, 14.1; p<0.01), number of children (≥2) (OR: 5.4, 95% CI: 2.5, 11.7; p<0.001) and family history of melasma (OR: 5.7, 95% CI: 2.7, 11.9; p<0.001). However, in the adjusted multivariate models, melasma was significantly associated with age ≥35 years (OR: 4.3, 95% CI: 1.6, 11.4; p<0.01) and being married (OR: 4.8, 95% CI: 1.1, 21.7; p<0.01). Similarly, individuals with a family history of melasma had higher odds of melasma (OR: 8.1, 95% CI: 2.9, 23.1; p<0.01) (Table 5). In multivariate models with just melasma patients, we found that the odds of metabolic syndrome were higher in females with melasma compared with males (OR: 1.2, 95% CI: 0.3, 4.9; p=0.84), even though this association was not statistically significant. However, males were more likely to have high triglyceride levels compared with females (OR: 8.6, 95% CI: 1.6, 47.5; p=0.014); this association was statistically significant.

**Table 5:** Table showing unadjusted and adjusted logistic regression models for factors associated with melasma, Mumbai, India The estimates are odds ratios and their 95% confidence intervals. ^a^Included separated/divorced in this group. *p<0.05, **p<0.01.

Variables	Unadjusted models	Adjusted models
	Odds ratio (95% confidence intervals)	Odds ratio (95% confidence intervals)
Age groups (years)		
18-34	Reference	Reference
≥35	5.8 (2.9, 11.4)**	4.3 (1.6, 11.4)**
Gender		
Male	Reference	Reference
Female	2.1 (1.1, 3.9)*	1.7 (0.7, 4.1)
Marital status		
Never married	Reference	Reference
Married^a^	6.0 (2.6, 14.1)**	4.8 (1.1, 21.7)*
Number of children		
None	Reference	Reference
One	2.3 (0.9, 5.5)	0.9 (0.2, 3.3)
≥2	5.4 (2.5, 11.7)	1.3 (0.4, 4.5)
Socio-economic status		
Lower/upper lower	Reference	Reference
Lower middle	1.2 (0.5, 2.6)	0.8 (0.3, 2.3)
Upper middle	0.9 (0.4, 1.9)	0.5 (0.2, 1.4)
Family history (first-degree relatives)		
No	Reference	Reference
Yes	5.7 (2.7, 11.9)**	8.1 (2.9, 23.1)**
BMI categories		
Normal/underweight	Reference	Reference
Overweight	0.5 (0.2, 0.9)	0.5 (0.2, 1.3)
Obese	0.7 (0.3, 1.6)	0.4 (0.1, 1.3)
Sun exposure		
Less than one hour	Reference	Reference
1-2 hours	0.3 (0.3, 1.1)	1.6 (0.6, 4.2)
>2 hours	0.9 (0.4, 2.4)	2.2 (0.7, 7.0)
Metabolic syndrome		
No	Reference	Reference
Yes	0.8 (0.4, 1.6)	0.5 (0.2, 1,4)

**Figure 1:**
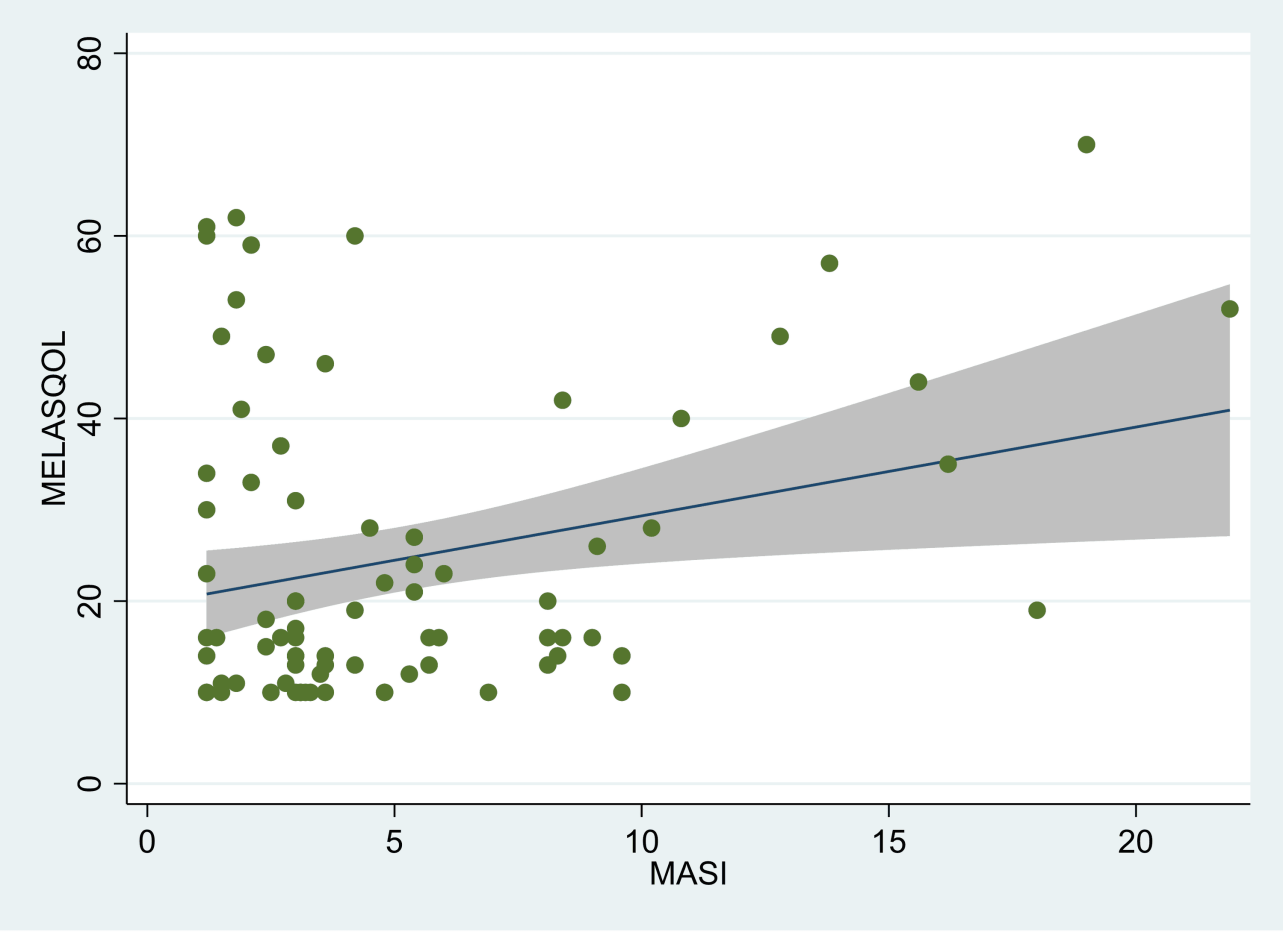
Scatter plot between MASI and MELASQOL scores in all melasma patients, Mumbai, India. MASI: Melasma Area and Severity Index score, MELASQOL: Melasma Quality of Life. The figure also shows the fitted line along with the confidence intervals.

**Figure 2:**
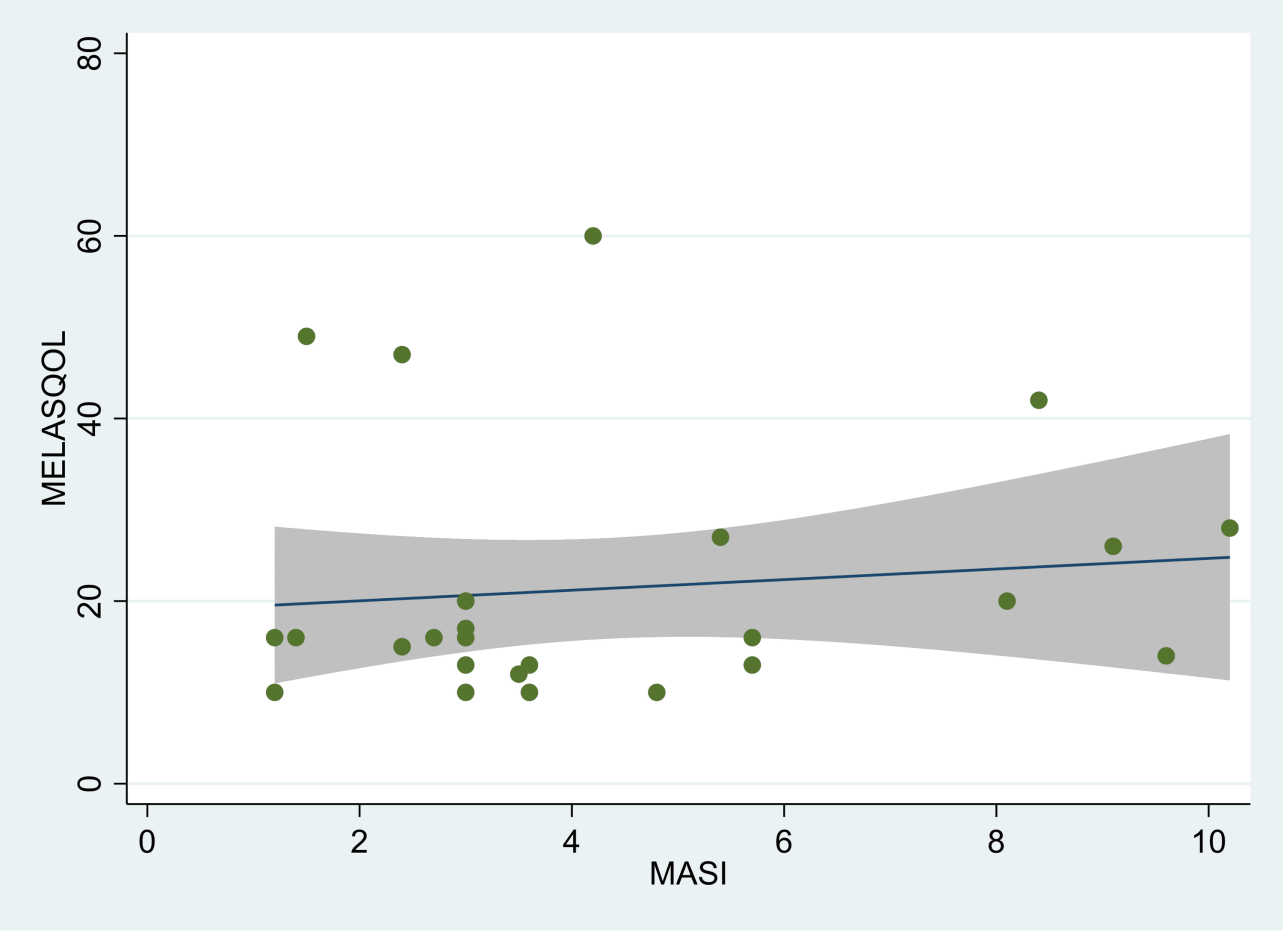
Scatter plot between MASI and MELASQOL scores in male melasma patients, Mumbai, India. MASI: Melasma Area and Severity Index score, MELASQOL: Melasma Quality of Life. The figure also shows the fitted line along with the confidence intervals.

**Figure 3:**
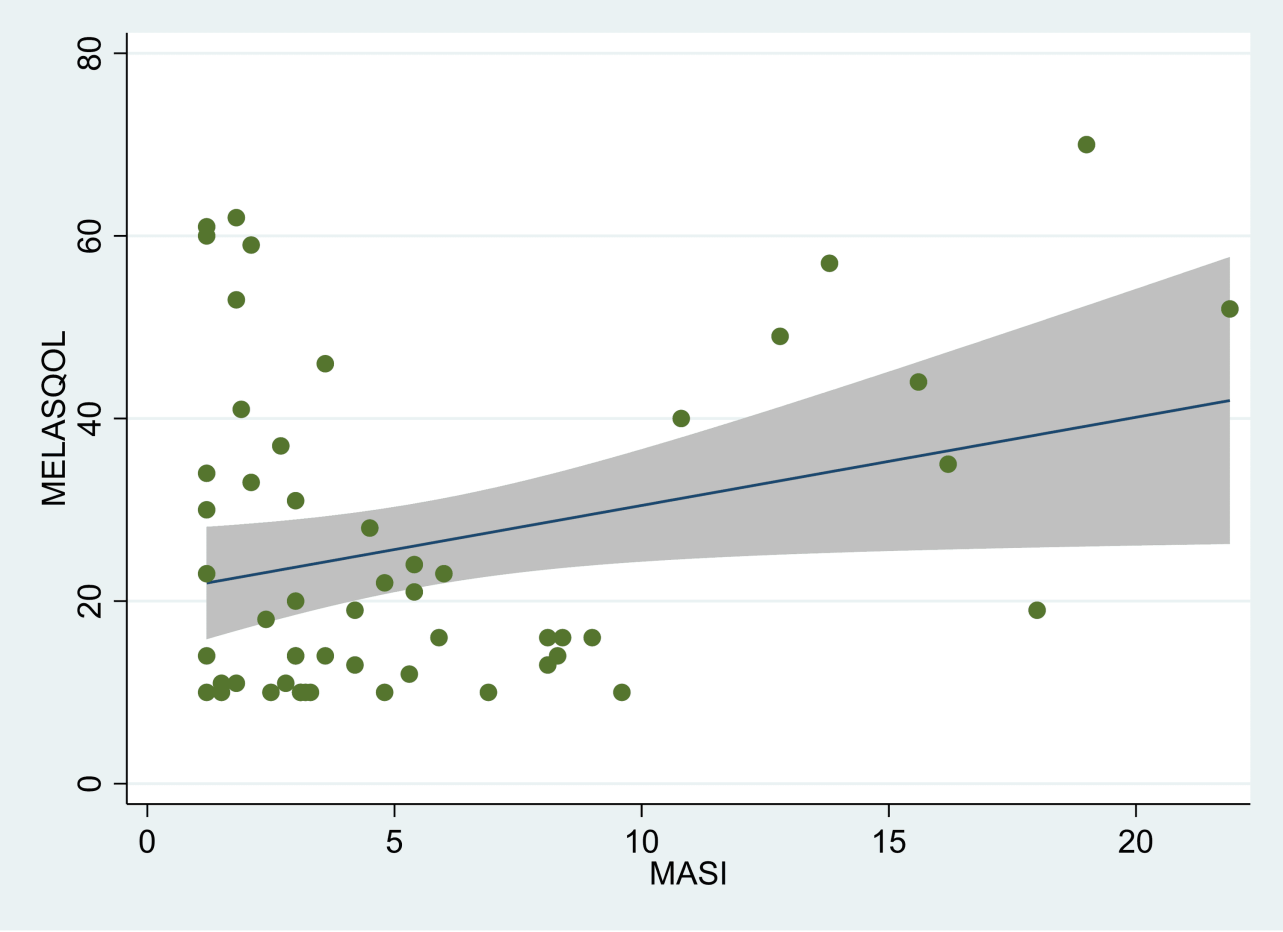
Scatter plot between MASI and MELASQOL scores in female melasma patients, Mumbai, India. MASI: Melasma Area and Severity Index score, MELASQOL: Melasma Quality of Life. The figure also shows the fitted line along with the confidence intervals.

There was a significant correlation between the MASI score and MELASQOL in individuals with melasma (r=0.27; p=0.017) (Figure 1). However, on stratification by gender, we found that the correlation was statistically significant only in females (r=0.28; p=0.04) (Figure 2). Among males, however, the correlation co-efficient was not statistically significant (r=0.12; p=0.573) (Figure 3). There was no significant correlation between duration and quality of life (r=-0.10; p=0.36) or age and quality of life (r=0.11; p=0.33). In the linear regression model, we found that with each unit increase in the MASI score, the MELASQOL increased significantly (estimate: 0.97, 95% CI: 0.18, 1.77; p=0.017), indicating a worsening of quality of life with an increase in the MASI scores. In females, the regression estimate was statistically significant (estimate: 0.97, 95% CI: 0.04, 1.89; p=0.04), whereas in males, this estimate was not statistically significant (estimate: 0.58, 95% CI: -1.52, 2.68; p=0.57).

## Discussion

In our study population, the factors associated with melasma were age ≥35 years, a family history of melasma, and being married. Furthermore, there was no significant difference in the likelihood of metabolic syndrome between these two groups. In melasma cases, in general, there was no significant difference in demographic and clinical characteristics between males and females. However, males had significantly higher levels of triglycerides compared with females. The association between melasma severity and quality of life was significant only in females.

Melasma may have a multifactorial etiology, including genetic, hormonal, exposure to sun (ultraviolet rays), and other inflammatory factors [8,47,48]. Previous studies have suggested that family history is an important factor associated with the occurrence of melasma. Platsidaki and colleagues reported a family history in 38% of melasma patients, whereas Martin and co-workers found a higher proportion of 46% [49,50]. However, Achar and Rathi found that 33% had a family history of melasma [13]. In our study, about 53% had a family history of melasma. Older age (≥35 years) was also an independent risk factor for melasma in our study. Previous studies have also reported onset of melasma in the later third to fourth decade, including increased severity with older ages-this may be due to the cumulative effect of risk factors over time [13,51,52]. Marital status was also associated with melasma in our study population. Though previous studies have identified hormones, age, parity, and the number of children as important risk factors for melasma, including its severity, these have not specifically addressed "marital status" as an independent risk factor [14,36,51,53]. Thus, this is an additional independent association found in our study. In our study, female melasma cases had a higher number of children compared with male cases; however, in the overall study population, the number of children was not significantly associated with melasma. The other important risk factor associated with melasma is sun exposure. Studies have reported that exposure to sunlight may be associated with the aggravation of melasma, and ultraviolet radiation is considered to be an important factor in the pathogenesis and the severity of melasma [11,14,54-56]. In our study, even though the odds were higher in those exposed to sunlight for a longer time, it was not statistically significant. This may be due to the small number of individuals having longer durations of sun exposure, while the majority of them had exposure for less than one hour daily; however, the direction of the association was similar to that seen in the literature.

The other factors that were compared were biochemical parameters and metabolic syndrome in cases and controls. In general, we did not find any difference between these biochemical parameters between cases and controls. Previous literature has discussed the contributory role of fatty acid metabolism and oxidative stress in melasma, even though some authors have suggested that there is no direct evidence that lipid metabolism contributed to the occurrence of melasma [24,27]. Silva and Steiner found that 29.3% of patients with melasma had features of MetS and suggested that metabolic factors may be considered in the management of melasma [57]. Other authors have studied the lipid profile in patients with melasma and controls. A previous study has shown that high-density lipoproteins (HDL) were significantly lower in melasma patients compared with controls, whereas levels of serum triglycerides, total cholesterol, and low-density lipoproteins (LDL) were significantly higher in the melasma group [26]. These authors included 50 melasma patients and 20 controls for their study. Another study by Ghassemi and colleagues found that LDL was significantly higher in the melasma group compared with controls, whereas there was no significant difference in the levels of blood sugars, liver enzymes, and other parameters of the lipid profile [25]. This study has only included female melasma patients and controls. In our study, we did not find any significant difference in the levels of triglycerides, HDL, fasting blood sugar, or HbA1c levels between the cases and controls. Furthermore, in our study, there was no significant association between MetS and melasma. Even though the prevalence of MetS was nearly similar to a previous melasma study and also similar to the prevalence reported in the Indian population [57-59]. Other authors have also cautioned about the overemphasis of metabolic syndrome in melasma [27]. We did not find any significant difference in the prevalence of MetS even between female and male cases with melasma. However, we did find that male melasma patients were significantly more likely to have high levels of triglycerides--this can be explored further in future studies.

The other aspect we evaluated is the quality of life in patients with melasma. Previous studies have provided mixed results on the association of the severity of melasma and quality of life [34]. While some studies have shown a significant correlation between the severity of melasma and the quality of life scores, others have not shown any significant correlation [38,60-62]. We found that there was a significant correlation between the severity and quality of life only in females; this was not seen in male melasma patients. This may indicate that female patients may be affected more due to cosmetic appearance and pigmentation. Other factors associated with quality of life have also shown mixed results. While some studies report a correlation between age or duration of melasma and quality of life, others did not find a significant correlation for these parameters [62-64]. We did not find any significant correlation between age or duration of melasma and quality of life in these patients.

Limitations

This was a case-control study. The findings should be interpreted as an association, and we are not claiming causality in this study. Furthermore, as with other case-control studies, there may be a recall bias--it is quite likely that cases may be more likely to recollect history related to exposure compared with controls [65]. There are a lot of other factors that have been evaluated in melasma, such as hormonal levels, antibodies, or oxidative biomarkers, which have been presented in the literature [21,66-69]. Other authors have also evaluated other lipid profile parameters in their research [25]. In our study, we focused on the metabolic parameters that were a part of MetS. We have also studied the demographic and risk factors (including MetS) associated with melasma, compared features in male and female melasma patients, and assessed the quality of life in them, thus providing a comprehensive assessment of melasma patients.

## Conclusions

Thus, in this study, we found that age ≥35 years, being married, and family history (in first-degree relatives) were significantly associated with melasma. There was no significant association between MetS and melasma in our study population; in fact, the prevalence of MetS was slightly lower in the cases compared with controls (even though it was not statistically significant). There was no significant difference in the levels of triglycerides, HDL, fasting blood sugar, or HbA1c between cases and controls. There was no significant difference in these parameters between male and female melasma patients, except for serum triglycerides. Male melasma patients were significantly more likely to have high levels of triglycerides--this may be explored further in future studies. Even though the prevalence of MetS was higher in female melasma cases compared with males, the difference was not statistically significant. The correlation between the severity of melasma and quality of life was significant only in female melasma patients.
